# Retraction: Research on anomaly detection and operational status evaluation methods for smart electricity meters based on hybrid deep learning

**DOI:** 10.1371/journal.pone.0355030

**Published:** 2026-08-03

**Authors:** The PLOS One Editors

The *PLOS One* Editors retract this article [1] due to concerns about the addition of a large number of citations to the Reference list without editorial approval. Many references are irrelevant to the article or corresponding cited statements, including the References 18, 19, 23, 54, 60, 62, 64, 71, 73, and 76, and Reference 277 is retracted. These issues raise concern about the validity and integrity of the article.

JunqX did not agree with the retraction. YZ, JunyZ, and ZB either did not respond directly or could not be reached.
